# Effect of fiber-matrix adhesion on the creep behavior of CF/PPS composites: temperature and physical aging characterization

**DOI:** 10.1007/s11043-016-9294-z

**Published:** 2016-02-12

**Authors:** M. H. Motta Dias, K. M. B. Jansen, J. W. Luinge, H. E. N. Bersee, R. Benedictus

**Affiliations:** 1 Faculty of Aerospace Engineering, Delft University of Technology, Kluyverweg 1, 2629HS Delft, The Netherlands; 2 Department of Design Engineering, Delft University of Technology, Landbergstraat 15, 2628CE Delft, The Netherlands; 3 TenCate Advanced Composites BV, G. van der Muelenweg 2, Postbus 9, 7440AA Nijverdal, The Netherlands

**Keywords:** Viscoelastic creep behavior, Physical aging, CF/PPS composites, Time aging–time superposition, Time temperature superposition principle (TTSP)

## Abstract

The influence of fiber-matrix adhesion on the linear viscoelastic creep behavior of ‘as received’ and ‘surface modified’ carbon fibers (AR-CF and SM-CF, respectively) reinforced polyphenylene sulfide (PPS) composite materials was investigated. Short-term tensile creep tests were performed on ±45° specimens under six different isothermal conditions, 40, 50, 60, 65, 70 and 75 °C. Physical aging effects were evaluated on both systems using the short-term test method established by Struik. The results showed that the shapes of the curves were affected neither by physical aging nor by the test temperature, allowing then superposition to be made. A unified model was proposed with a single physical aging and temperature-dependent shift factor, $a_{T,te}$. It was suggested that the surface treatment carried out in SM-CF/PPS had two major effects on the creep response of CF/PPS composites at a reference temperature of 40 °C: a lowering of the initial compliance of about 25 % and a slowing down of the creep response of about 1.1 decade.

## Introduction

Over the last decades, fiber-reinforced polymeric matrix composites (PMCs) have become the materials of choice in many structural applications involving aircraft, aerospace, military vehicles, automobiles, civil infrastructure, medical devices, and sporting equipment. Their enhanced specific strength and stiffness, among other major advances, have enabled this class of materials to be used in place of traditional metallic alloys. Commonly, thermoset (TS) resins are used to constitute the polymeric matrix; however, there is a growing trend to replace TS with high-performance thermoplastics (HPTPs). Although HPTP can be bought at prices comparable to their TS counterpart, their processing costs are higher. Typically, HPTP processing involves higher temperatures and pressures than TS, a downside mitigated by the reduced handling, shorter processing time (enabling larger series production) and the improved performance characteristics of its composites, compared to TS-based composites. Yet two of the best performing HPTPs, polyphenylene sulfide (PPS) and polyetherimide (PEI), do not offer a sufficient performance to be potential candidates to replace traditional metallic alloys in most structural and load bearing applications (Luche 2013). An alternative way to increase the performance of these materials further is to tailor the composite fiber-matrix adhesion. This has been shown directly to affect the end-use component static short-term mechanical properties (Madhukar and Drzal 1991a, 1991b, 1992; Carnevale et al. 2012). The rising interest in fiber-HPTP adhesion tailoring has encouraged researchers worldwide to modify existing TS-compatible fiber sizings and optimize fiber pre-treatments processes. Although the key role performed by fiber-matrix adhesion in the static short-term mechanical performance of PMCs is widely recognized, its importance on the static long-term mechanical performance of PMCs, referred to as creep and stress relaxation in this paper, has not been extensively addressed. Regardless of the characteristics of the fiber-matrix adhesion, all PMCs show the time-dependent behavior that is peculiar to the viscoelastic nature of a polymeric matrix material (Shaw and MacKnight 2005; Papanicolaou and Zaoutsos 2010). Nevertheless, the range and rate at which these processes occur may be affected by the fiber-matrix adhesion quality of these materials, justifying a study of this topic.

As most PMCs are designed for long-term service lives, typically expressed in decades, measuring their viscoelastic responses over their entire lifetimes is reasonably impractical. As a consequence, many studies have been conducted and published on accelerated test methods that are used to characterize the viscoelastic behavior of PMCs (Gates et al. 1997; Goertzen and Kessler 2006; Barbero 2010). Accelerated methods use momentary creep-recovery data and models to predict the long-term behavior of the material. A successful example of these models is the time–temperature superposition principle (TTSP). Although the background theory for the TTSP was originally developed for neat solid polymers (Ward and Sweeney 2004), several researchers have expanded its use to PMCs (e.g., Schapery 1974; Ferry 1980; Gates et al. 1997; Goertzen and Kessler 2006; Barbero 2010).

The goal of the research reported here was to investigate the influence of the fiber-matrix adhesion on the linear viscoelastic behavior of ‘as received’ (AR) and ‘surface modified’ (SM) woven-carbon fiber (CF) reinforced polyphenylene sulfide (PPS) composites. Creep tests were performed on composite specimens using elevated temperature, sub-glass transition, $T_{g}$, as the accelerator factor, to assess the viscoelastic behavior of these materials. Physical aging effects were also characterized using the momentary test method established by Struik (1978). This work is part of a research program aimed at developing strategies to tailor fiber-matrix interfaces in high performance TP composites for different applications.

## Background

A brief review of the theoretical aspects related to the effect of fiber-matrix adhesion, physical aging and temperature, in PMC systems is presented in this section.

### Fiber-matrix adhesion

The market for continuous high performance CF embedded in polymeric resin is still dominated by TS resins. As a consequence, most of the commercially available CF is surface-treated for TS systems. Over the last two decades, however, this described scenario has been forced to change, as TPs are gradually displacing TSs in an ever-increasing number of applications. Regardless of the type of polymeric matrix, the effects of their interaction with the CF reinforcement on the static long-term mechanical performance have received little attention in the literature. In the 1990s, Chang et al. (1993) investigated the effect of the interphase region on creep response and creep rupture of CF reinforced a TP-based PMC, with different surface treatments and/or sizings. The authors showed that for the studied systems, the interfacial adhesion between fibers and matrix affects neither the creep response, characterized by the shape of the creep curves, nor the degradation rate of the creep rupture strength. Moreover, it was shown that, although the creep rupture strength was dependent of the fiber-matrix adhesion quality, fiber-matrix adhesion that yields good static mechanical properties does not necessarily generate good creep rupture strength. Subramanian et al. (1995) investigated the interphase effects on the creep response of CF reinforced a TS-based PMC, with different surface treatment and/or sizings. Unlike the findings of Chang, Subramanian et al. showed that the fiber-matrix interphase plays a significant role in the creep response of the studied PMCs. The type of sizing, TP- or TS-based, was shown to influence the creep response, with TP-based sizing resulting in a composite material more susceptible to creep. As for the extra treatment carried out on already treated CF, no further improvement of the creep response was achieved. Neither of these works, however, considers the interphase as an independent viscoelastic constituent when interpreting the obtained results. Li and Weng (1996) investigated the effect of a viscoelastic interphase of finite concentration on the overall creep and stress–strain relationships of a glass fiber (GF) reinforced ED-6 composites. The authors showed that, while the axial tensile creep and stress–strain behavior are not effectively affected by both the interphase properties and the interphase volume concentration, the creep and stress–strain responses of the GF/ED-6 composite are strongly dependent on these two parameters when load is applied at other directions. When the interphase was softer than the matrix, the creep strength and load-carrying capacity of the composite was greatly reduced by its presence. This reduction was shown to be very sensitive to both concentration of the interphase and its properties.

Carnevale (2014) has recently extensively studied the fiber-matrix adhesion mechanisms taking place at the interphase between CF, with different surface treatments, and PPS composites, the same composite system discussed in this paper. The author proposes possible interactions between the CF and PPS by relating the results obtained from analyses of the CF’s sizing and its surface, using Fourier-Transform Infrared Spectroscopy (FT-IR), X-ray photoelectron spectroscopy (XPS) and Brunauer–Emmet–Teller (BET) adsorption measurements, to those obtained for the composite’s static short-term mechanical properties. According to the author, the presence of sizing, TS-compatible, in AR-CF/PPS does not lead to an improvement of the composite’s mechanical performance, but instead, to a deterioration. It was shown that the non-reacted sizing present at this fiber surface hindered mechanical interlocking of PPS on the CF’s surface, and formed a low modulus interlayer between the CF and the PPS. Neither of these occurs when AR-CF undergo an additional surface treatment to become SM-CF, due to the greater capability of PPS to interlock mechanically with PPS matrix which is the main reason for the SM-CF/PPS better static short-term mechanical performance, when compared to AR-CF/PPS. Although the work of Carnevale successfully proposes possible relations between the CF-PPS interactions and the resulting static short-term mechanical properties of the composite, it does not adequately explain the resulting long-term mechanical performance of the composite. These results, together with the different results obtained by Chang et al. and Subramanian et al., described above, highlighted the need for a better understanding of the role of fiber matrix adhesion in the long-term mechanical properties of PMCs. In this work the interphase is not considered to be an independent viscoelastic material, but a composite constituent that affects the viscoelastic behavior of the polymeric matrix, and consequently that of the studied PMC.

### Temperatures effects

Polymer materials and their composites deform, or flow, over time when a constant load is applied. This deformation, or flow, results from the molecular motion of the polymer chains in an attempt to minimize localized energy (Papanicolaou and Zaoutsos 2010). With an increase of temperature, the frequency of the molecular regrouping will increase, speeding up the creep process, accordingly. The physical interpretation of this temperature dependence is that all retardation times, $\tau_{i}$, for the molecular processes responsible for the linear viscoelastic behavior of glassy polymers are affected to the same degree by a change in temperature, $T$, according to Eq. (1),

1 $$ a_{T}=\frac{\tau_{i}(T_{\mathrm{ref}})}{\tau_{i}(T)}, $$

where $\tau_{i}$ is the $i$th retardation time, $a_{T}$ is the temperature shift factor and $T_{\mathrm{ref}}$ the reference temperature.

The $a_{T}$ is a basic property of a particular material. Several semi-empirical models that describe the dependence of $a_{T}$ on temperature have been proposed in the literature (Aniskevich et al. 2012). Their use, however, depends upon the temperature range covered in the tests. For temperatures below $T_{g}$, $a_{T}$ can be fitted using a log-polynomial model:

2 $$ \log a_{T}(T)=b_{0}+b_{1}T+b_{2}T^{2}, $$

where $b_{0}$, $b_{1}$, and $b_{2}$ are parameters determined by fitting experimentally obtained data to Eq. (2). Note that, due to its quadratic nature, the accuracy of Eq. (2) is mostly strictly limited to the measured range.

The approach above is referred to in the literature as the time–temperature superposition principle (TTSP) (Findley et al. 1989), and its general form is described by:

3 $$ S(t;T)=S_{\mathrm{ref}}(a_{T}t;T_{\mathrm{ref}}), $$

where $S(t; T)$ is a generic representation for any compliance component and $S_{\mathrm{ref}} (a_{T} t; T)$ is the representation for the compliance at the reference curve.

### Physical aging effects

**Fig. 1:**
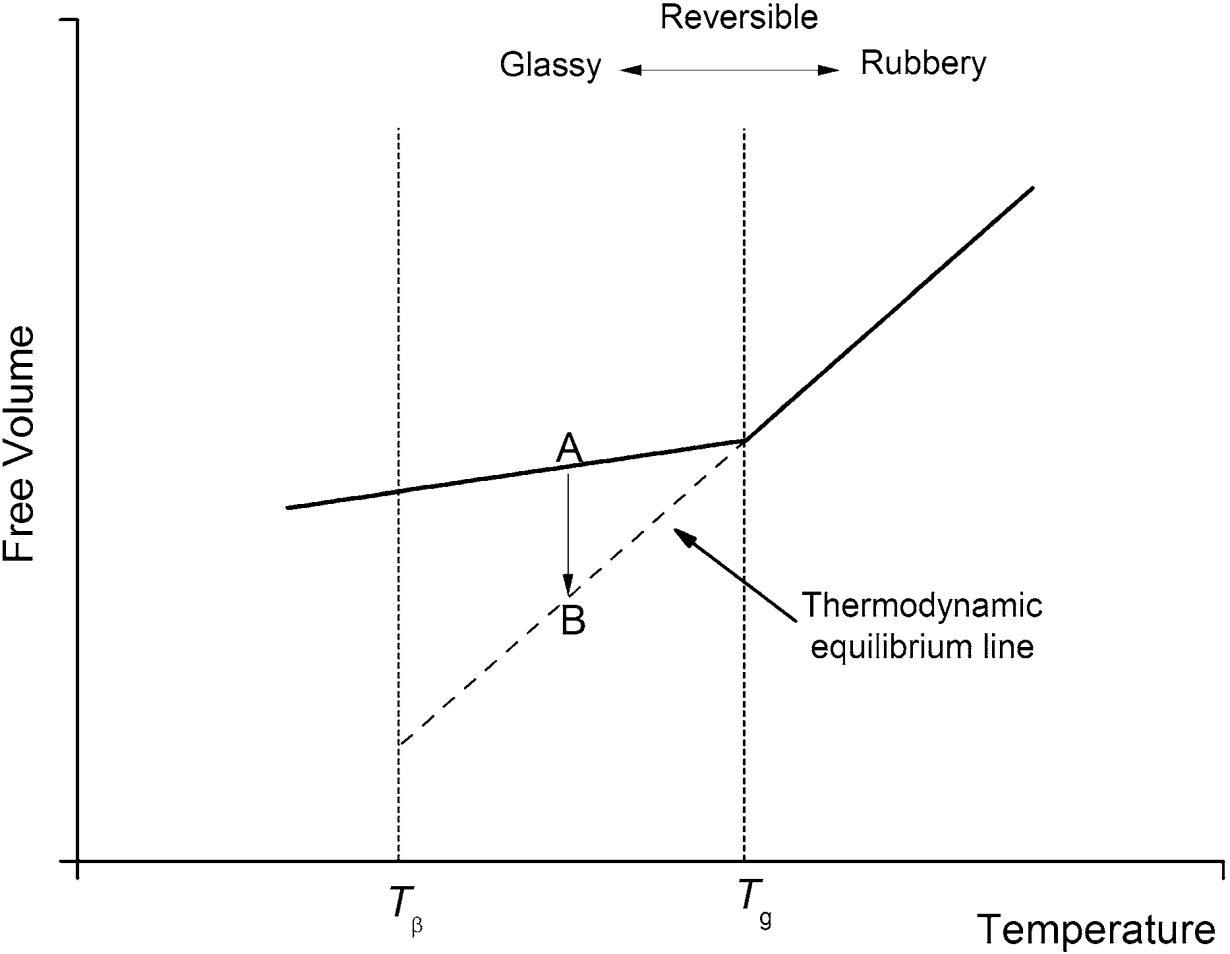
Physical aging of polymers explained from the free-volume concept

At a molecular level, the physical aging process and its influence on the viscoelastic properties of polymer glasses is a manifestation of a slow reduction in free volume, i.e., the unoccupied portion of the specific volume, over time after a polymeric material has been quenched to below its $T_{g}$. The time at which this process occurs is referred to as the aging time, $t_{e}$. This volume change associated with isothermal physical aging from a given point A to B is shown in Fig. 1 (Struik 1978).

As $t_{e}$ progresses, a series of momentary creep tests can be run to obtain the creep response of a material. Since ‘physical aging’ is the only type of aging considered in this study, the terminologies ‘aging’ and ‘physical aging’ are used interchangeably and refer to the same aging process.

The physical aging behavior of semi-crystalline polymers greatly differs from that of amorphous polymers. In semi-crystalline polymers, Struik (1987a, 1987b, 1989a, 1989b), Krishnaswamy et al. (2003) and Vlasvelda et al. (2005) have shown that physical aging process still remains at temperatures well above the measured bulk $T_{g}$ of the polymeric material. An explanation for this extended temperature window involves a range of different amorphous phases, in which molecular and segmental mobilities depend upon their proximity to a rigid crystalline region. Further from a rigid crystalline phase, molecular and segmental mobility increases and the $T_{g}$ decreases towards the bulk $T_{g}$, that is, the mobile-amorphous phase. On the other hand, close to the rigid crystalline phase, the segmental mobility is restricted and the $T_{g}$ increases, that is, the rigid-amorphous phase. The aging behavior below the lowest (bulk) $T_{g}$, the mobile-amorphous phase, is similar to the behavior of neat amorphous polymers.

One important point to keep in mind when discussing the physical aging effects on PMCs is that, unless the fibers and their sizing somehow interact with the polymeric resin, or the viscoelastic response of the interphase plays a significant role, the PMC aging will be the same as that of the neat resin (McKenna 2011). In agreement with this statement, the work reported here is built on the hypothesis that, the low modulus interlayer present in AR-CF/PPS system is capable of slightly altering the aging response of the CF/PPS system under investigation.

Since both $t_{e}$ and $T$ affect the time constant $\tau_{i}$, a general shift factor, $a_{T,te}$, can be defined by modifying Eq. (1) to obtain Eq. (4) given below (Sullivan et al. 1993):

4 $$ a_{T;t_{e}}(T;t_{e})=\frac{\tau_{i}(T_{\mathrm{ref}};t_{e,\mathrm{ref}})}{\tau _{i}(T;t_{e})}, $$

where $t_{e},_{\mathrm{ref}}$ is the aging time at which the reference curve was defined.

The general $a_{T,te}$ is related to the individual’s temperature $a_{T}$ below $T_{g}$, and the aging time shift factor, $a_{te}$, by Eq. (5),

5 $$ \log a_{T,te}(T,t_{e})=\log a_{T}+ \log a_{te}. $$

Struik (1978) has shown that, for thermorheologically simple materials undergoing isothermal physical aging, Eq. (3) can be modified to include aging time effects by rewriting it as

6 $$ S(t;T,t_{e})=S_{\mathrm{ref}}(a_{T,te}t;T_{\mathrm{ref}},t_{e,\mathrm{ref}}). $$

## Experimental procedures

### Test materials and specimen configuration

Two material systems were investigated for this study, ‘as received’ and ‘surface modified’ carbon fibers (AR-CF and SM-CF, respectively) reinforced polyphenylene sulfide (PPS) composites. The high performance PPS thermoplastic resin was ‘FORTRON 0214^®^’ fabricated by Celanese and provided by TenCate Advanced Composites (TenCate) in the form of 80 μm thick film. The nominal PPS melt temperature, $T_{m}$, was 285 °C. The CFs used were T300J 40B 3K Polyacrylonitrile (PAN) based, standard modulus, fabricated by Toray Soficar (Toray) and provided by TenCate in the form of 5-harness satin fabric with two different surface treatments being:

(i) As provided by Toray to TenCate, referred to as ‘as received’ (AR) in this work. The fibers are surface-treated and surface-coated with 1 % weight Bisphenol A diglycidylether type surface coating by the original suppliers, Toray.
(ii) Woven AR-CF fabric which had received an additional industrial surface heat treatment carried out by TenCate—referred to as ‘surface modified’ (SM) in this work. Optimized surface treatment of fibers may either produce chemical bonds on their surface (leading more interaction with the matrix) or roughen the surface (increasing mechanical keying between the resin and fiber), or do both, which leads to more adhesion with the matrix. The heat treatment carried out by TenCate aims to roughen the surface of CF. Nevertheless, due to confidentially reasons, no details about the heat treatment are disclosed.

The composite panels, measuring 600 by 600 mm, were produced by hand stacking of the woven fabric CF and PPS resin film and subsequent thermopressing. The stacking sequences used were $[0,90]_{4s}$, where $[0,90]$ represents one fabric ply. The layers were stacked so that they were symmetric with respect to the middle plane and that the warp side faces the outside. The quality of the composite panels was assessed by ultrasonic single through transmission inspection with an automated C-scanner of dimensions 3 by 1.5 m produced by Midas Inc. using water probes as coupling media. The C-Scan indicated that all produced panels had a good consolidation, with absence of any delaminations or voids. The fiber volume fraction of each produced laminate was estimated by weight measurement of specimens cut out from each laminate. Both composites had a CF volume fraction of about 50 %. Differential Scanning Calorimetry (DSC) was used to estimate the degree of crystallinity in the matrix in the presence of the fibers in the both CF/PPS composites. The crystallinity of the two composites was found to be 57 % and 54 % for AR-CF/PPS and SM-CF/PPS, respectively, using a value of heat of fusion of a 100 % crystalline sample of PPS of 76.5 J/g (Spruiell and Janke 2004). Within the experimental error, these values can be considered to be the same.

**Fig. 2:**
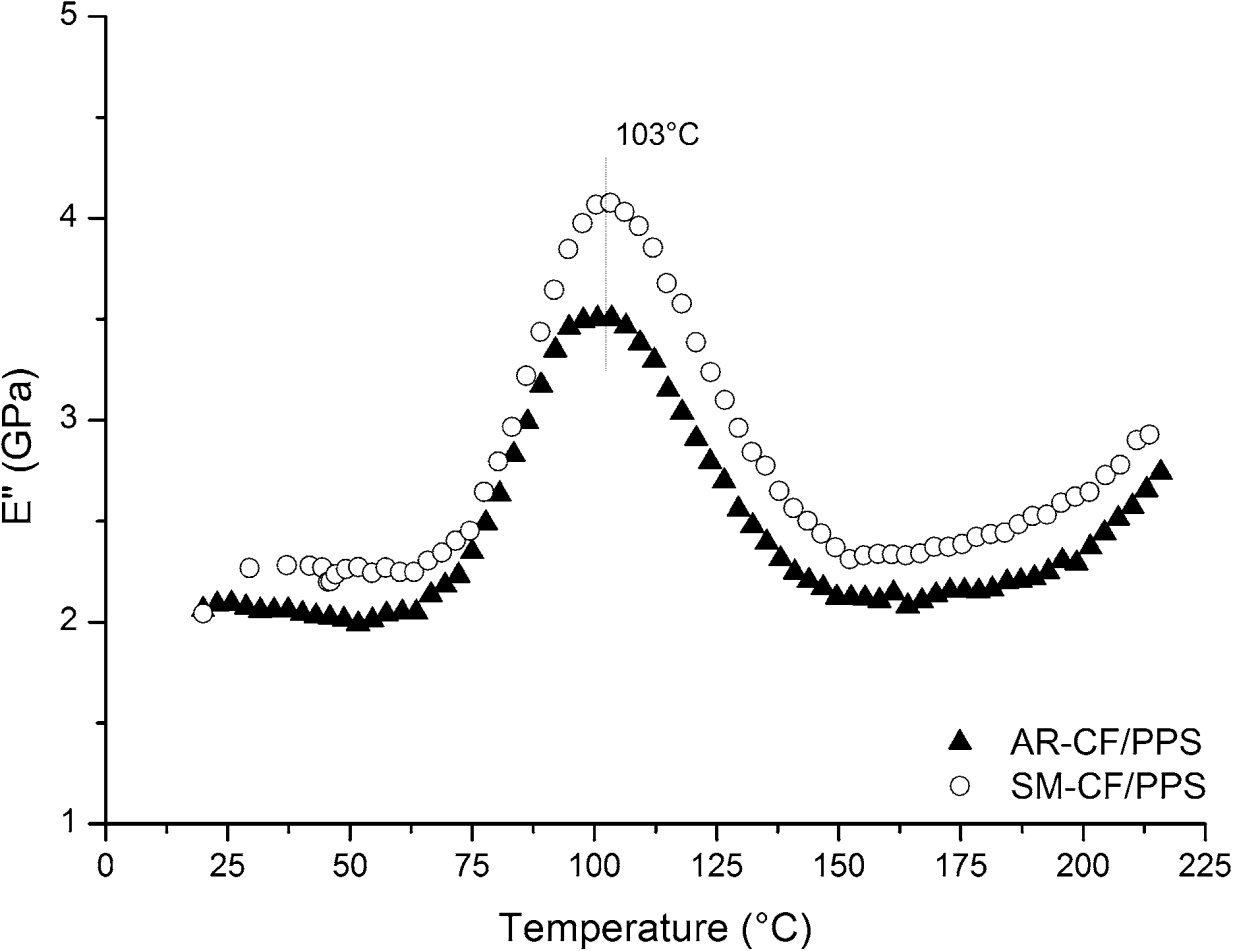
Loss modulus curve of AR-CF/PPS and SM-CF/PPS

The bulk $T_{g}$ for both materials were found to be around 103 °C and defined as the temperature associated to the maximum of the loss modulus peak, measured using a dynamic mechanical analyzer (DMA) at 1 Hz and 2.5 °C/min, and presented in Fig. 2.

Diffusion of the sizing molecules in a PPS matrix is a possible mechanism in the studied system. Desio and Rebenfeld (1992) report a significant decrease in the bulk $T_{g}$ of AS4 CF/PPS composites and of a commercial Phillips prepreg when sizing diffusion occurs in the PPS matrix. The decrease was explained as plasticization of the PPS due to the sizing molecules. The fact that no significant difference in bulk $T_{g}$ was measured in the DMA tests suggests that no, or very limited, sizing diffusion had occurred in the bulk PPS matrix. Nevertheless, from Fig. 2 it can be seen that, despite the bulk $T_{g}$ of both materials being the same, the curve corresponding to AR-CF/PPS is slightly shifted to lower temperatures, hence the onset of the glass transition is about 7 °C lower comparing to the onset of the $T_{g}$ for SM-CF/PPS.

Test specimens, measuring approximately 250 mm in length, in the load direction, and 25 mm in width, were cut from 2.5 mm thick laminated panels at an angle of ±45° to the fibers using a water cooled diamond saw. The size of the specimen is in accordance with the ASTM Specification D3518/D3518M. Despite the fact that the ±45° angle-ply is not the most suitable lay-up to use for a structural component, the in-plane shear response produced by performing tensile tests on such specimens is widely known as an interphase sensitive test (Madhukar and Drzal 1991a; Carnevale 2014). Since carbon fibers are time-independent materials, using the time-dependent form of the classical laminate theory, given in Jones (1975) for a single lamina under plane stress conditions, the only time-dependent compliance term in the compliance matrix that will play a role in creep for a $[0,90]$ woven fabric ply is the shear ($S_{66}$), obtained from in-plane shear test, see Eq. (7):

7 $$ [S]=\left [ \textstyle\begin{array}{c@{\quad}c@{\quad}c} S_{11} & S_{12} & 0\\ S_{12} & S_{22} & 0\\ 0 & 0 & S_{66}(t) \end{array}\displaystyle \right ], $$

where subscripts 1, 2 are the material coordinates referenced to the directions along and transverse to the fiber, respectively, and the subscript 66 refers to shear.

Paper tabs were glued to the specimen’s surface longitudinal ends to avoid slippage. Strain gages, type KFG-5-120-C1-11, from Kyowa, were used for the strain measurements. In total, 3 strain gages were used for each tested specimen; 2 strains gages were mounted vertically aligned back-to-back in the center of the specimen, so that any possible buckling of the specimen would be detected; 1 strain gage was mounted longitudinally aligned in the center of the specimen.

In order to separate load induced strain from thermal strain, temperature compensation gauges were mounted on another specimen placed close to the test specimen. This specimen remained unloaded throughout the test. The thermal apparent strain measured in this specimen was then used to correct the measured strain in the loaded specimen.

### Test equipment

All creep tests were performed using a 10 kN Zwick 1445 tensile tester machine equipped with a convection oven. To obtain a better accuracy between the temperature displayed at the oven and the real specimen temperature, a thermocouple was attached to the center region of the tested specimen surface. The temperature of the specimen during each set of aging and creep tests was maintained constant throughout the test. Prior to testing, the specimens were dried for at least 24 hours at 60 °C in a vacuum oven. After each creep test, specimens were stored for 2 days in a desiccator to prevent moisture absorption, rejuvenated by heating to 115 °C for 30 minutes followed by a quenching to the test temperature, and then subjected to a new set of isothermal physical aging and creep testing at a different sub-$T_{g}$ temperatures of 40, 50, 60, 65, 70 or 75 °C (±0.5 °C). The physical aging process was defined as starting immediately after a specimen reached the desirable test temperature.

### Creep testing

In order to obtain the in-plane shear compliance response, test specimens were loaded in tension at an angle of ±45° to the fiber orientation. To ensure that all the tests were performed within the linear viscoelastic range, a preliminary study was carried out. Test specimens of AR-CF/PPS and SM-CF/PPS were rejuvenated, quenched and subjected to sets of momentary creep tests at in-plane shear stresses ranging from 3 to 30 MPa at a temperature of 70 °C. The in-plane shear creep compliance response of the ±45° specimens is given by Eq. (8). Since in-plane shear is the only type of stress response discussed in this paper, the symbol $S$ instead of $S_{66}$ is used throughout this paper,

8 $$ S=\frac{2A(e_{x}-e_{y})}{P}, $$

where $S$ is the in-plane shear creep compliance, $P$ is the axial load applied on the specimen, $A$ is the cross-sectional area of the specimen, $e_{x}$ is strain along the loading direction, and $e_{y}$ is the strain along the transverse direction.

If the applied load places the material outside the linear viscoelastic range, the momentary compliance response will vary depending on stress level, indicating that the material is in the nonlinear range. In this work, the linear–nonlinear threshold stress was taken to be a stress level at which the compliance obtained at the lowest in-plane shear stress, 3 MPa, would vary by 10 %. Although linearity conditions were not tested at 75 °C, it was assumed that the results obtained at 70 °C would also hold true at 75 °C. Nevertheless, caution should be taken with this assumption, as temperature has been reported to play a major role in the linear–nonlinear threshold stress for viscoelasticity of PMCs (Aniskevich et al. 2012).

**Fig. 3:**
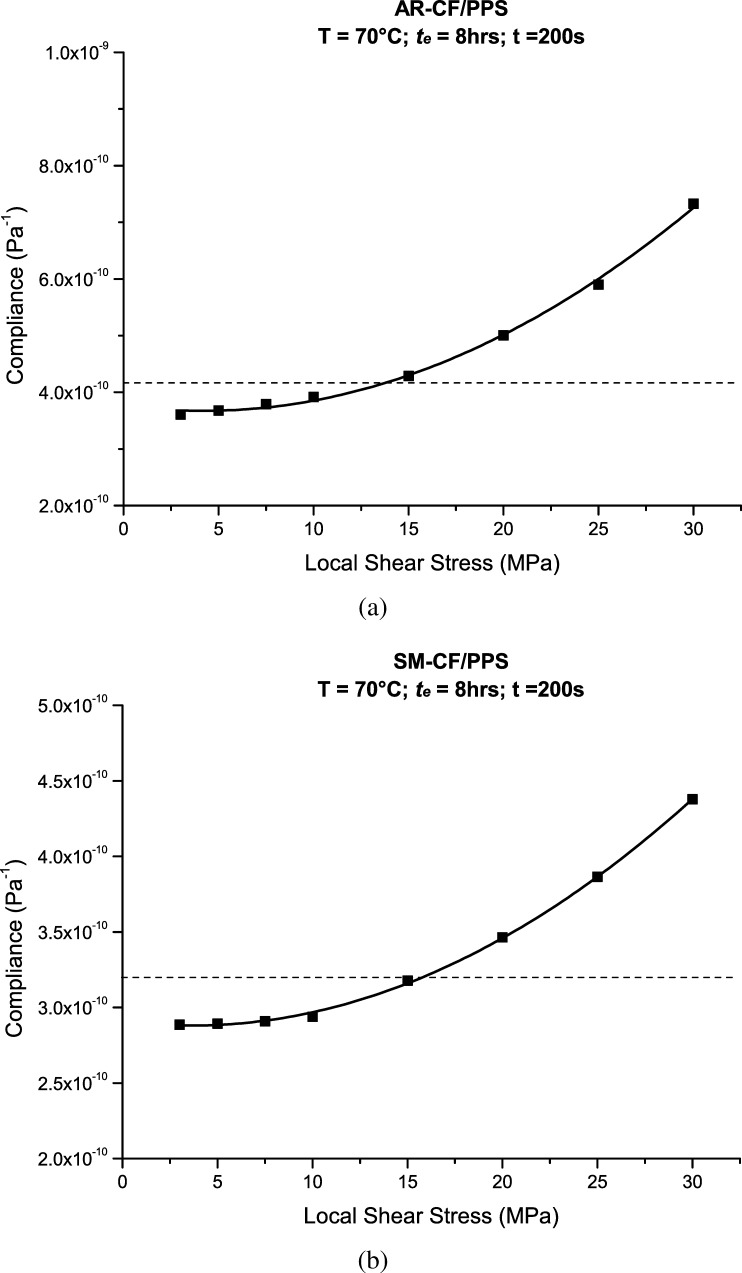
Isochronous curve of (**a**) AR-CF/PPS and (**b**) SM-CF/PPS

The isochronous curves of the AR-CF/PPS and SM-CF/PPS composites obtained after following the above procedure are shown in Fig. 3. The solid lines fitted through the data points are just guidelines placed there to help with the data visualization. The same applies for the dotted lines, which represent the linear–nonlinear threshold in-plane shear stresses.

Based on the methodology described above, and to promote comparability between AR-CF/PPS and SM-CF/PPS, the same in-plane shear stress level of 7 MPa was chosen for this study.

The physical aging times selected for starting each creep segment were 4, 8, 24, 48 and 96 hours and the duration of each creep tests, $t$, was chosen to be $0.125t_{e}$, thus assuring momentary data. After each creep segment, the specimen was unloaded and allowed to recover until the start of the next creep test. To account for any remaining residual strain due to a lack of full recovery, the strain measured in the creep segment was corrected by subtracting the extrapolated recovery strain from the prior creep curve (Struik 1978).

**Fig. 4:**
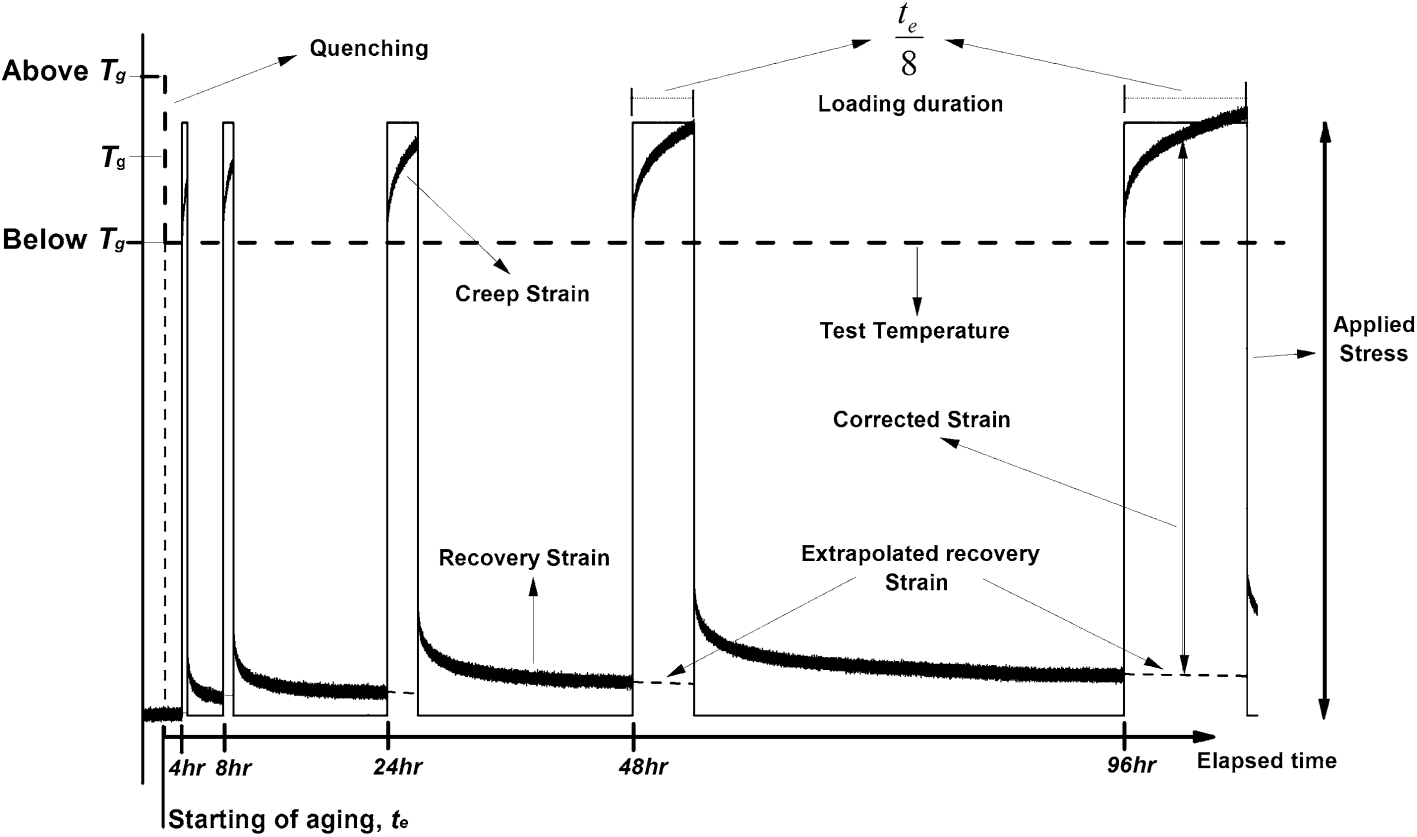
Illustration of the sequence of creep and recovery tests for determining aging effects

All the procedures described above were repeated at all tested temperatures for both AR-CF/PPS and SM-CF/PPS; a schematic representation of a single test temperature is shown in Fig. 4.

Replicate tests were performed for 3 test temperatures and showed typical deviation of about 3 %. Since these tests results did not deviate significantly, subsequent replicate tests were not carried out due to practical issues.

## Results and discussion

**Fig. 5:**
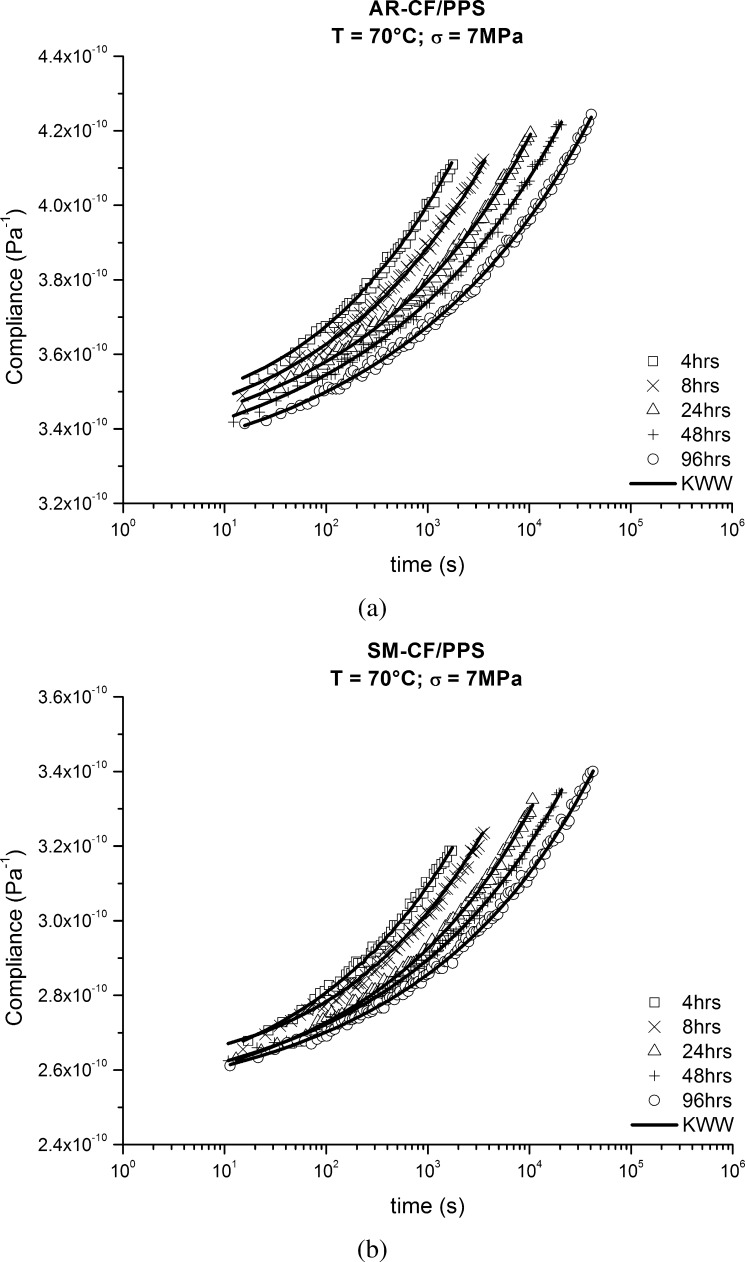
Momentary creep compliance as a function of time (in semi-log scale) at 70 °C, 7 MPa and different aging times for (**a**) AR-CF/PPS and (**b**) SM-CF/PPS

The momentary creep compliance versus time curves for AR-CF/PPS and SM-CF/PPS specimens at 70 °C, and different aging times, are shown in Fig. 5. All the individual compliance curves were regressed to the three parameter fit model, first proposed by Kohlrausch (1854) and later used by Williams and Watt (1969) and now known as Kohlrausch–Williams–Watts (KWW) function:

9 $$ S(t)=S_{0}e^{(\frac{t}{\tau(t_{e})})^{\beta}}, $$

where $S_{0}$ is the initial compliance, $\beta$ is the shape parameter, $t$ is the time elapsed since the application of the load, and $\tau (t_{e})$ is the physical aging dependent retardation time.

Physical aging leads to a decrease in free volume in polymeric materials, which, consequently, causes a reduction in segmental mobility, an increase in the degree of packing, stiffening, and a slowdown in creep response. The curves for the tested materials shown in Fig. 5 confirmed these expectations. The creep curves shifted towards longer times as the aging times increased. The time shift between the 4 and 96 hrs curves in Figs. 5(a) and 5(b) is about 1.0 and 0.8 decades, respectively.

The KWW parameters used to characterize each of the individual momentary curves shown in Fig. 5 are given in Table 1.

**Table 1:** Momentary curves parameters for both AR-CF/PPS and SM-CF/PPS at 70 °C

$t_{e}$ (hrs)	AR-CF/PPS aged 96 hrs, 7 MPa			SM-CF/PPS aged 96 hrs, 7 MPa		
	$S_{0}$	*τ* (s)	*β*	$S_{0}$	*τ* (s)	*β*
4	3.26 × 10^−10^	8.96 × 10^9^	0.23	2.43 × 10^−10^	5.16 × 10^5^	0.23
8	3.25 × 10^−10^	2.57 × 10^8^	0.22	2.49 × 10^−10^	9.25 × 10^5^	0.24
24	3.28 × 10^−10^	5.79 × 10^7^	0.22	2.46 × 10^−10^	2.21 × 10^6^	0.23
48	3.23 × 10^−10^	1.59 × 10^7^	0.20	2.47 × 10^−10^	5.23 × 10^6^	0.22
96	3.20 × 10^−10^	3.21 × 10^7^	0.19	2.47 × 10^−10^	9.42 × 10^6^	0.21

**Fig. 6:**
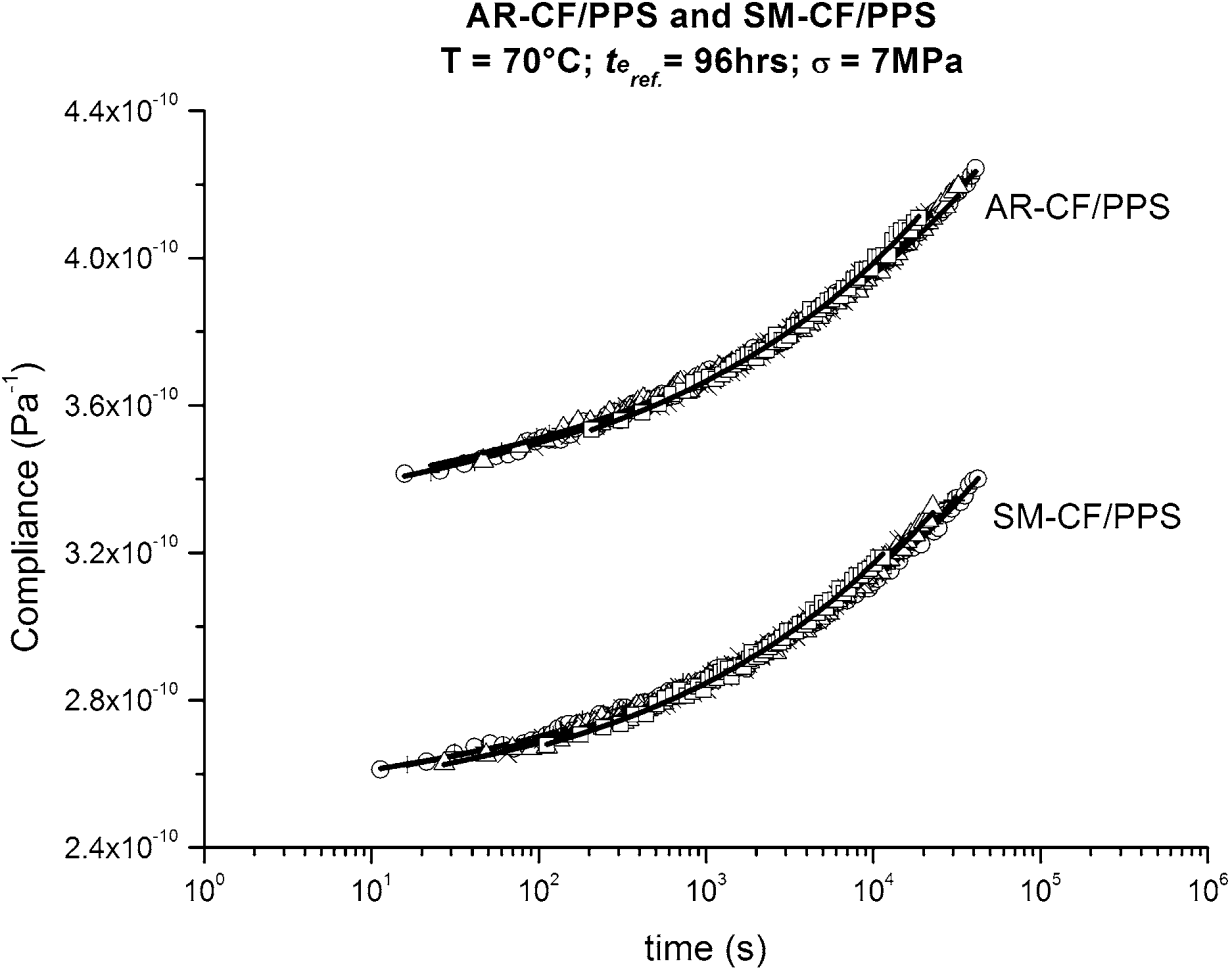
Momentary master curves for AR-CF/PPS and SM-CF/PPS obtained by using time–aging time superposition on momentary compliance curves from Fig. 5; in both cases, the reference physical aging time is 96 hours

For isothermal physical aging, individual momentary curves can be superposed using horizontal shifting in a log-time scale. Time–aging time superposition was used to generate a single master curve of all the data at a reference aging time of 96 hours for both AR-CF/PPS and SM-CF/PPS. An algorithm based on minimizing the mismatching in compliance using a least squares method was used to generate the aging shift factors, $a_{te}$. The resultant curves of the shifting procedures are presented in Fig. 6.

It can be seen from Fig. 6 that the additional fiber surface treatment carried out in SM-CF improves the adhesion between the CF and the PPS matrix, resulting in a reduction in the initial compliance, $S_{0}$, at 70 °C of about 25 % compared to AR-CF/PPS. It is assumed that this lower $S_{0}$ for SM-CF/PPS, mostly elastic response due to the applied load, is credited to the greater capability of the PPS matrix to interlock mechanically with the rougher SM-CF surface, agreeing with the findings of Carnevale (2014).

**Fig. 7:**
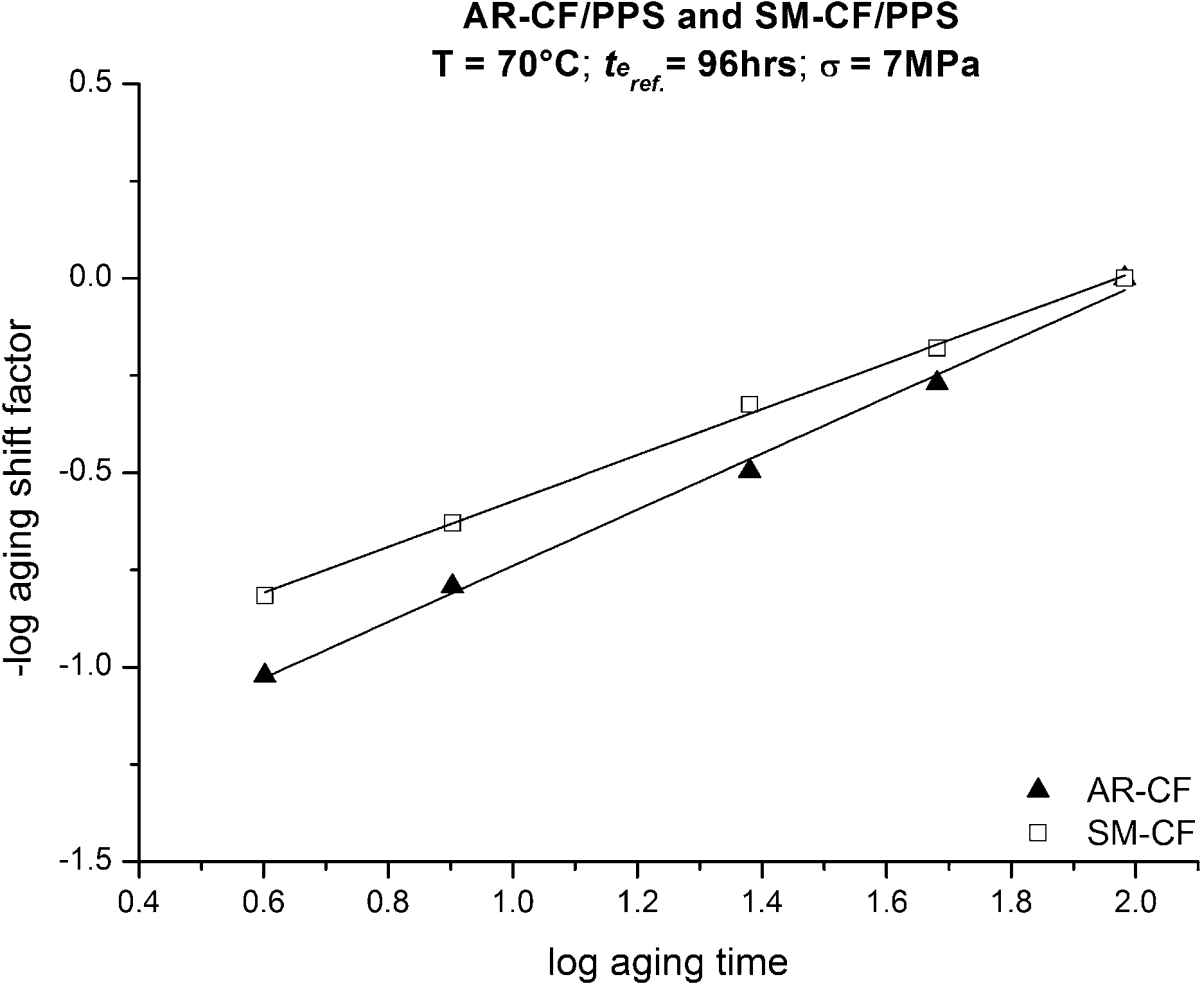
Aging shift factors obtained using time–aging time superposition for AR-CF/PPS and SM-CF/PPS at 70 °C and 96 hours reference curve

The obtained $a_{te}$ versus the logarithmic $t_{e}$ at 70 °C are shown in Fig. 7, here the data points are fitted using linear regression.

Similar curves and trends to those presented in Figs. 5, 6 and 7 were obtained for all the other tested temperatures. The slope of the straight lines presented in Fig. 7 is the temperature dependent aging shift rate, $\mu_{e}$, given by Eq. (10),

10 $$ \mu_{e}(T)=\frac{-d\log a_{te}}{d\log t_{e}}. $$

**Fig. 8:**
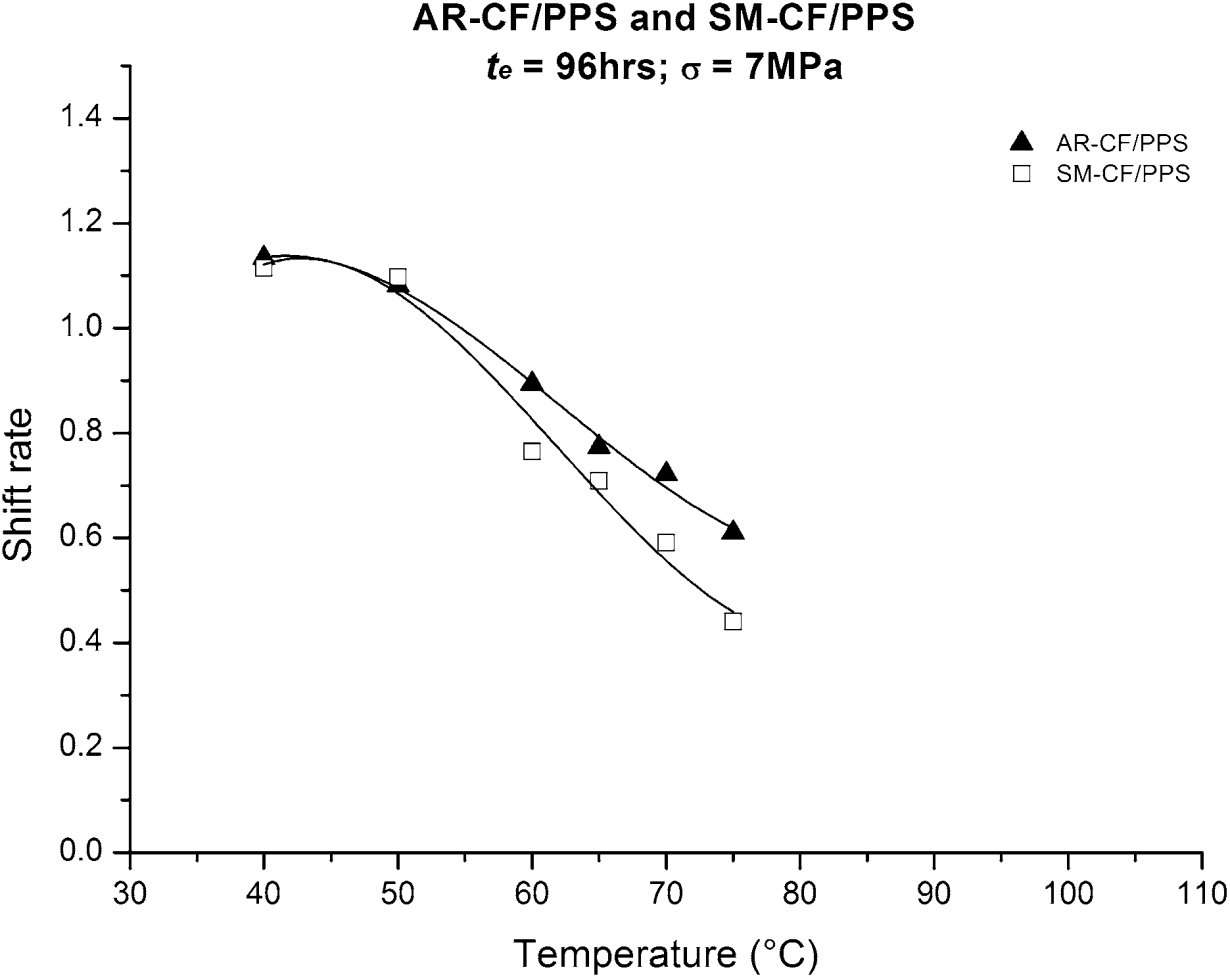
Aging shift rate, $\mu_{e}$, as a function of temperature for AR-CF/PPS and SM-CF/PPS

For the 70 °C tests shown in Fig. 7, the $\mu_{e}$ for AR-CF/PPS and SM-CF/PPS are 0.72 and 0.60, respectively. The $\mu_{e}$ values obtained by repeating all of the procedures discussed above for all tested temperatures are shown in Fig. 8.

From Fig. 8 it can be seen that, for both tested materials, $\mu_{e}$ decreases with an increase in temperature. Note that, due to the semi-crystalline nature of the PPS polymeric matrix used, above the measure bulk $T_{g}$, approximately 103 °C, $\mu_{e}$ is expected to be very low but not to vanish completely. At the measured bulk $T_{g}$, the material is approaching thermodynamic equilibrium. Nevertheless, as stated in Struik (1987a, 1987b, 1989a, 1989b), Krishnaswamy et al. (2003), and Vlasvelda et al. (2005), in semi-crystalline materials, aging can persist even at temperatures above bulk $T_{g}$. The fact that the obtained $\mu_{e}$ for both materials were nearly the same up till approximately 50 °C, and then diverged can possibly be attributed to the difference in the $T_{g}$ in the interphasial region presented in the AR-CF/PPS system. The shift on the onset of the glass transition of AR-CF/PPS, shown in the DMA results of Fig. 2, supports this hypothesis, even though no evidence of sizing diffusion in the PPS matrix could be measured in the bulk $T_{g}$. The curves shown in Fig. 8 suggest that the non-reacted sizing presented in AR-CF diffuses in the PPS matrix near the interphase region, locally decreasing the $T_{g}$ enough to affect the creep response, yet not enough to affect the bulk $T_{g}$ of the AR-CF/PPS overall material. Aging processes in polymers and PMCs are directly related to the distance between the temperature at which the aging process is taking place and the $T_{g}$. The free volume reduction, caused by aging, accelerates with proximity to the $T_{g}$. A smaller free volume translates into slower creep response, a greater $a_{te}$ leads to a collapse of the individual curves (4, 8, 24 and 48 hrs) on the 96 hrs reference curves, and, consequently, a greater $\mu_{e}$. This assumption is confirmed by the data shown in Fig. 8 for temperatures above 50 °C. At lower temperatures, however, both the tested materials were still subjected to deformation within the low molecular motion glassy region, which gave nearly the same creep response for each material over time, and consequently, the same $\mu_{e}$.

The characterization of aging in terms of the mathematical expression implies that the only parameter in Eq. (9) that changes with aging is the retardation time, $\tau(t_{e})$, given by Eq. (11),

11 $$ \tau(t_{e})=\tau(t_{e,\mathrm{ref}}) \biggl( \frac{1}{a_{te}}\biggr), $$

where $t_{e,\mathrm{ref}}$ is the reference aging time, $a_{te,\mathrm{ref}}=1$, and the aging shift factor is given by Eq. (12),

12 $$ a_{te}=\biggl(\frac{t_{e,\mathrm{ref}}}{t_{e}}\biggr)^{\mu_{e}(T)}. $$

Thus to describe the momentary creep compliance at any elapsed aging time, $t_{e}$, at an isothermal condition, the required parameters are: the initial compliance, $S_{0}$, the shape parameter, $\beta$, the shift rate, $\mu_{e}$, and the retardation time, $\tau$, at a reference aging time, $t_{e,\mathrm{ref}}$.

**Fig. 9:**
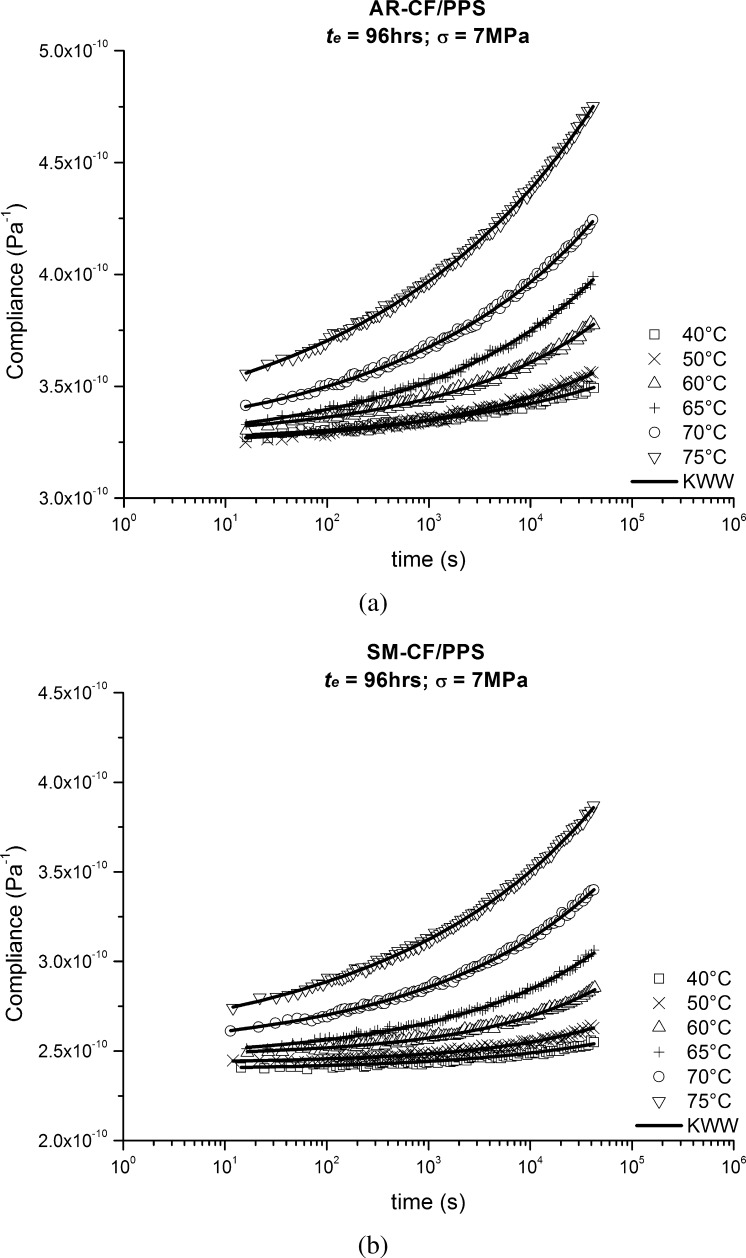
Compliance as a function of time (log-scale) for (**a**) AR-CF/PPS and (**b**) SM-CF/PPS time–aging time master curves (96 hrs curves) at different temperatures

Compliance of the 96 hours aged curves versus time for AR-CF/PPS and SM-CF/PPS specimens at all the tested temperatures are shown in Fig. 9. The 96 hours reference curves were chosen to represent the time–aging time master curve. This choice was based on the fact that these were the curves with more data points and therefore likely to be the best in terms of the mechanical conditioning of the test specimen. All of the individual 96 hours compliance curve was regressed to the KWW function.

The KWW parameters used to characterize each of the individual momentary curves in Fig. 9 are given in Table 2. The expected fact that higher temperatures will accelerate molecular rearrangements in viscoelastic materials is described in Table 2, column $\tau$, where it can be seen that an increase in temperature resulted in a shortening of the retardation time $\tau$. Note that creep compliance develops slower for a larger retardation time.

**Table 2:** 96 hrs curves’ parameters and temperature shift factors, $a_{T}$, for both AR-CF/PPS and SM-CF/PPS

*T* (°C)	AR-CF/PPS aged 96 hrs, 7 MPa				SM-CF/PPS aged 96 hrs, 7 MPa			
	$S_{0}$	*τ* (s)	*β*	$a_{T}$	$S_{0}$	*τ* (s)	*β*	$a_{T}$
40	3.23 × 10^−10^	3.79 × 10^9^	0.23	1.0	2.40 × 10^−10^	5.96 × 10^8^	0.30	1.0
50	3.23 × 10^−10^	3.03 × 10^8^	0.26	1.3	2.43 × 10^−10^	5.90 × 10^7^	0.35	8.2
60	3.26 × 10^−10^	5.73 × 10^7^	0.27	12.1	2.47 × 10^−10^	1.63 × 10^7^	0.33	160.6
65	3.22 × 10^−10^	3.27 × 10^7^	0.24	40.4	2.45 × 10^−10^	1.35 × 10^7^	0.26	931.5
70	3.20 × 10^−10^	3.21 × 10^7^	0.19	219.0	2.47 × 10^−10^	9.42 × 10^6^	0.21	11503.6
75	3.14 × 10^−10^	1.31 × 10^7^	0.15	2375.1	2.43 × 10^−10^	4.65 × 10^6^	0.16	119867.5

The 96 hrs curves for AR-CF/PPS and SM-CF/PPS of all the tested temperatures were collapsed to obtain single overall material master curves using TTSP. The collapse was done successfully using the 40 °C curves as the reference curves and by horizontally shifting all the remaining curves. The same algorithm used in the aging superposition was used to generate the temperature shift factors, $a_{T}$; see Table 2, column $a_{T}$.

**Fig. 10:**
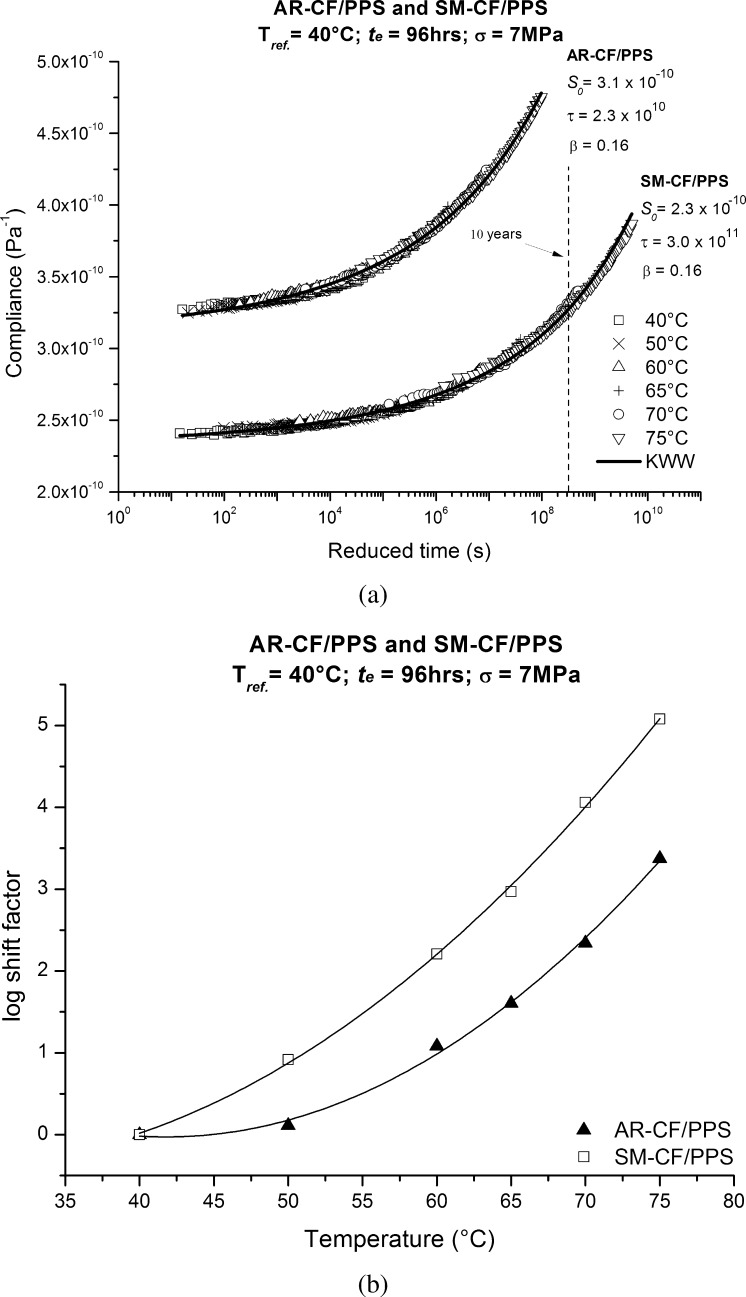
(**a**) Creep master curves of time–aging time master curves (96 hrs curves) specimens for AR-CF/PPS and SM-CF/PPS; (**b**) temperature shift factors as a function of temperature for AR-CF/PPS and SM-CF/PPS

The TTSP allowed the obtained overall master curves, along with the temperature shift factor versus temperature curves, to be used to predict momentary response at any temperature within the tested range; both these curves are presented in Fig. 10.

An analysis of the KWW parameters in Fig. 10(a) showed that the initial compliance, $S_{0}$, and the retardation time, $\tau$, changed depending to the type of CF/PPS system, while the shape parameter, $\beta$, remained the same. As mentioned above, in the KWW equation, the $S_{0}$ is mostly related to the initial elastic response of the material, and is thus expected to be different. At the reference temperature of 40 °C, the greater capability of the PPS matrix to interlock mechanically to the SM-CF and, therefore, to improve the load transferring from the matrix to fiber, is translated into an approximately 25 % reduction in $S_{0}$ after the AR-CF underwent additional surface treatment to become SM-CF. Although this initial elastic response did not make a significant contribution to understanding the time-dependent creep response of the studied systems, the $S_{0}$ interpretation is very important since it constitutes a considerable fraction of the allowable total compliance in designing. For the parameters $\tau$ and $\beta$, the fact that both curves have the same $\beta$, yet different $\tau$, supports the hypothesis of the presence of a interphase region in AR-CF/PPS, locally lowering the $T_{g}$ and consequently, an accelerated creep response. If the curves in Fig. 10(a) are normalized with the initial compliance, $S_{0}$, so that the elastic effect ceases to play a role, it is possible to see that, with a horizontal shift of about 1.1 decade in the AR-CF/PPS master curve, both AR-CF/PPS and SM-CF/PPS curves collapse into a single master curve. For design purposes, this 1.1 decade means that, to reach the same compliance level, the SM-CF/PPS specimens require more time, i.e., 1.1 decade, than the AR-CF/PPS specimens. This 1.1 decade in time can be interpreted as a difference in $T_{g}$ of about 9.5 °C when the AR-CF/PPS shift factor vs. temperature curve in Fig. 10(b) is horizontally shifted to collapse onto the SM-CF/PPS shift factor vs. temperature curve.

The use of the unified model described in Eq. (6) allows predictions to be made outside the range of ages and temperatures for which data is available. It is important to remark, however, that prediction far outside the experimental time window (over more than three decades of time) is not recommended.

## Conclusions

In this experimental study, the influence of fiber-matrix adhesion on the linear viscoelastic creep behavior of ‘as received’ and ‘surface modified’ carbon fibers (AR-CF and SM-CF, respectively) reinforced polyphenylene sulfide (PPS) composite materials was investigated. As a preliminary step to obtain the overall time–temperature master curve, and to characterize the changes in the matrix dominated properties of the composite over storage time, physical aging studies were carried out using the short-term test method established by Struik. Short-term compliance curves were obtained for different physical aging times at different sub-$T_{g}$ temperatures. All the individual curves were modelled using the KWW equation.

As expected, the obtained curves showed an increase in retardation times, $\tau$, or stiffening, as the physical aging times became longer. The shape of the curves were not affected by physical aging, thus time–aging time superposition was successfully applied to obtain aging master curves using the aging shift factors, $a_{te}$, generated by an algorithm. Using the 96 hours compliance curves as representative of the time–aging time master curves, TTSP master curves were obtained by shifting these curves using a temperature shift factor, $a_{T}$, and the 96 hours at 40 °C curve as the reference curve. A unified model was proposed with a single physical aging and temperature dependent shift factor, $a_{T,te}$.

It was suggested that the surface treatment carried out in SM-CF/PPS had two major effects on the creep response of CF/PPS composites at the reference temperature of 40 °C: a lowering of the initial compliance, $S_{0}$, of about 25 % caused by the absence of the interlayer formed by the non-reacted sizing, presented in the AR-CF/PPS system, which hinders the PPS matrix to interlock mechanically with the CF surface; a slowing down of the creep response of about 1.1 decade caused absence of diffusion of the non-reacted sizing in the PPS matrix near the interphase region, which has as a consequence a local reduction in $T_{g}$, and an acceleration in the physical aging process.
